# The effect of Bushen Huoxue method in treating glaucoma: A protocol for systematic review and meta-analysis

**DOI:** 10.1097/MD.0000000000021156

**Published:** 2020-07-02

**Authors:** Liyuan Wang, Tianyang Yu, He Sun, Ruoxi Liu, Ye Liu

**Affiliations:** a Department of Ophthalmology, First Affiliated Hospital of Heilongjiang University of Chinese Medicine; b Heilongjiang University of Chinese Medicine; c Department of Ophthalmology, Second Affiliated Hospital of Heilongjiang University of Chinese Medicine, Harbin, Heilongjiang, China.

**Keywords:** Bushen Huoxue method, glaucoma, protocol, systematic review, traditional Chinese medicine

## Abstract

**Background:**

Glaucoma is a common ophthalmic neurodegenerative disease and the main cause of blindness, which seriously affects the life and work of patients, without more effective treatment for optic nerve damage. Bushen Huoxue (BSHX) method is a traditional Chinese medicine (TCM) therapy that has been widely used as an alternative therapy to treat optic nerve damage in glaucoma patients with growing beneficial effect evidence, however, there is no current systematic review has addressed its effect for glaucoma. This study will conduct a systematic review and meta-analysis of the currently published randomized controlled trials (RCTs) of BSHX method for the treatment of glaucoma, aim to assess the efficacy and safety of BSHX method for patients with glaucoma.

**Methods:**

We will thoroughly search literatures of RCTs related to BSHX method for glaucoma in PubMed, Medline, EMBASE, Cochrane Library, China National Knowledge Infrastructure (CNKI), VIP and Wanfang database and other databases from the establishment of the database to November 2019, with no language restriction. After reviewing the title, abstract and full text, 2 reviewers will independently select the study, extract the data, after assess the risk of bias, we will conduct a meta-analysis of the data extracted from the included RCTs, including total effective rate, intraocular pressure (IOP), visual acuity, visual field, TCM syndrome score, and adverse events. The meta-analysis will be performed using Review Manager 5.3 software and the results will be based on either random effects or fixed effects models, depending on the heterogeneity. Trial sequential analysis (TSA) and Grading of Recommendations, Development and Evaluate system (GRADE) will be conduct to evaluate the reliability and quality of evidence.

**Results:**

The results of the study will be published in a peer-reviewed journal, and provide a reasonable and high-quality evidence for the efficacy and safety of BSHX method for glaucoma.

**Conclusion:**

This study will be the first meta-analysis to evaluate the efficacy of BSHX method in the treatment of glaucoma comprehensively, and will to provide helpful evidence for the clinical treatment of this disease.

**Registration:**

PROSPERO CRD42020159897

## 1 Introduction

Glaucoma is a chronic neurodegenerative eye disease with complex pathogenesis and multiple factors, accompanied by visual impairment and visual field loss, which is one of the main causes of blindness worldwide.^[1,2]^ The prevalence of glaucoma among people aged 40 to 80 is about 3.54% globally and about 4% in the Asian population.^[3,4]^ It is estimated that the number of glaucoma patients worldwide will increase to 79.6 million by 2020 and exceed 100 million by 2040.^[5,6]^ With the increasing incidence of glaucoma year by year, this irreversible blinding disease, has become an important challenge for ophthalmologists. At present, intraocular pressure (IOP) is considered to be the main risk factor that triggers the optic nerve damage of glaucoma patients.^[7,8]^

The treatment of glaucoma is mainly focusing on protect visual function by reducing IOP.^[9]^ However, IOP is not the only factor responsible for visual impairment in glaucoma, many patients have continued visual impairment after IOP has been reduced or controlled within the normal range.^[10]^ For the protective treatment of optic nerve in glaucoma patients, most of the treatment methods are still in the experimental stage, the toxic and side effects of many drugs still limit their clinical application.^[11]^ In China, traditional Chinese medicine (TCM) has a significant advantage in controlling optic nerve damage in glaucoma patients, and fewer adverse reactions, which has a better protective effect on the loss of visual function after IOP is stabilized.^[12,13]^ Therefore, it is of great significance to study the mechanism of the effect of TCM on optic nerve protection in glaucoma.

According to TCM theory, kidney deficiency and blood stasis are a common syndrome of glaucoma, which the blood stasis caused by kidney deficiency obstructs the vessels of the eye and leads to blindness.^[14]^ Thus Bushen Huoxue (BSHX) method, a QiJuDiHuang-Wan-derived TCM formula is commonly and effectively used in China treating glaucoma, Bushen means tonifying kidney and Huoxue means activating blood circulation which can regulating patients’ kidney deficiency and blood stasis condition. It is composed of rehmannia, yam, dogwood, *Rhizome alismatis*, poria, moutan cortex, goji, chrysanthemum, *Salviae miltiorrhizae*, pseudo-ginseng and borneol. In clinical trials, it has shown that BSHX method can improve vision, visual field in primary angle-closure glaucoma, and improve visual evoked potential (VEP), retinal nerve fiber layer thickness in glaucomatous eyes after filtering surgery.^[15,16]^ In animal experiments it has demonstrated that BSHX method can up-regulating the expression of retinal NF-κB, down-regulating the expression of retinal Caspase-9, reduced IOP slightly and improved the ultrastructure of RGCs.^[17]^

However, data supporting the validity of this treatment are insufficient. Therefore, in this study, we will systematically collect clinical evidence of BSHX method in the treatment of glaucoma, and conduct a systematic review and meta-analysis to evaluate efficacy and safety of BSHX method as a treatment for glaucoma by integrating different outcomes from clinical studies.

## 2 Methods and analysis

### 2.1 Study registration

A protocol includes the detailed search strategy and data analysis method has been registered on the International Prospective Register of Systematic Reviews (PROSPERO) with registration number CRD42020159897, basing on the Preferred Reporting Items for Systematic Reviews and Meta-analysis Protocols (PRISMA-P) statement guidelines on April 28, 2020.^[18]^

### 2.2 Inclusion and exclusion criteria for study selection

#### 2.2.1 Types of studies

Only randomized, controlled trials (RCTs) to assess the effects of Bushen Huoxue method in the treatment of glaucoma will be included without language restriction. Others such as case reports, non-RCTs, reviews and other types of study will be excluded.

#### 2.2.2 Type of participants

We will include studies on patients have been diagnosed as glaucoma based on any recognized diagnostic criteria, with no restriction of age, race, gender, and profession.

#### 2.2.3 Type of Interventions

Interventions of BSHX method alone or BSHX method combined conventional treatment will be included. Control group can be any type of treatment but not treated by BSHX method. If the intervention group is BSHX method combined conventional treatment, the treatment in the control group should be consistent with the conventional treatment that in the intervention group.

#### 2.2.4 Type of outcome measurements

##### 2.2.4.1 Primary outcome

The primary outcome is total effective rate, based on the improvement of vision, visual field and VEP examination together.

##### 2.2.4.2 Secondary outcomes

Secondary outcomes include IOP, vision, visual field, TCM syndrome score and adverse events.

### 2.3 Study search

#### 2.3.1 Electronic searches

We will search the relevant studies of BSHX method in the treatment of glaucoma in PubMed, Medline, EMBASE, Cochrane Library, China National Knowledge Infrastructure (CNKI), VIP and Wanfang database from their respective inception dates to November 2019. There will be no language restrictions in the search of studies. Two reviewers will search and screen all the citations independently according to the search strategies. The detailed search strategies in CENTRAL database are presented in Table 1, similarly strategies will be applied in other databases.

**Table 1:**
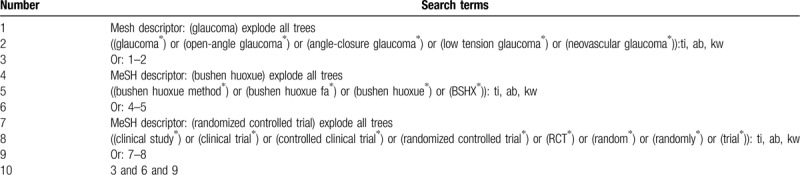
Search strategy for CENTRAL database.

#### 2.3.2 Other resources searches

We will search the ClinicalTrials.gov and Chinese Clinical Trial Registry (ChiCTR) to find potentially relevant clinical registrations, search Google to find other related literatures. A manual search will also be conducted at the library of Heilongjiang University of Traditional Chinese Medicine to avoid missing potential studies.

### 2.4 Data collection and analysis

#### 2.4.1 Selection of studies

**Figure 1:**
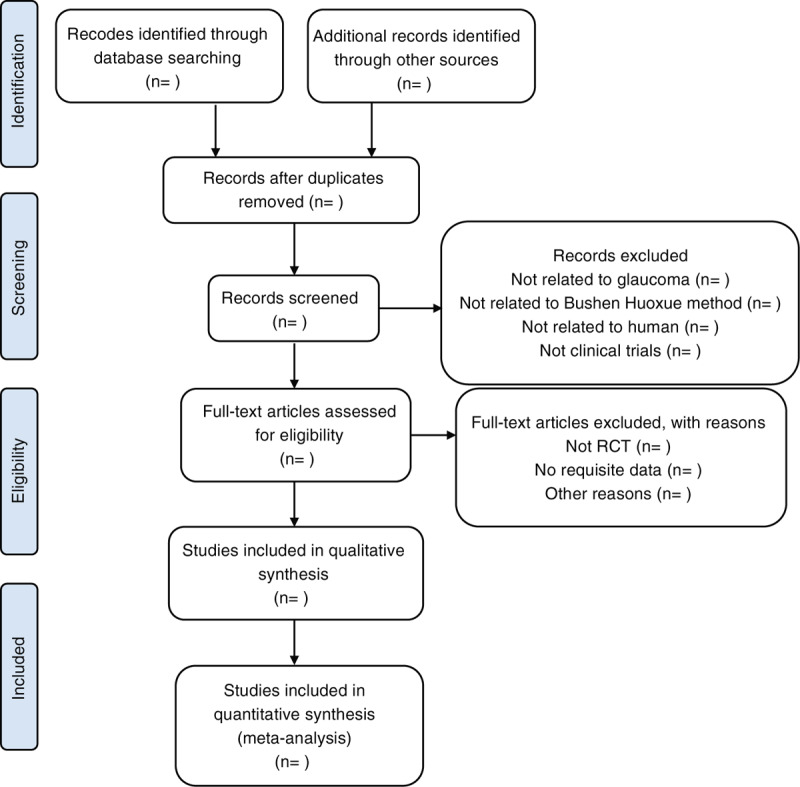
The flow chart of study selection process. RCT = randomized controlled trial. From: Moher D, Liberati A, Tetzlaff J, Altman DG, The PRISMA Group (2009). Preferred Reporting Items for Systematic Reviews and Meta-Analyses: The PRISMA Statement. PLoS Med 6(7): e1000097. doi:10.1371/journal. pmed1000097.

Two reviewers will independently run search strategy to identify potentially eligible studies. The results of the literature searches will be import into the literature manage software EndNote X7. After duplicates has been removed, the irrelevant studies will be removed by two reviewers after independently evaluate the titles and abstracts according to the inclusion and exclusion criteria. Then full text of the remaining studies will be read carefully to identify eligible studies. Any disagreement will be resolved through discussion between the 2 reviewers. When consultation fails to reach an agreement, arbitration will be resolved by discussion or consultation with a third reviewer. A flow chart according to PRISMA guidelines to illustrate the whole selection process is shown in Figure 1.

#### 2.4.2 Data extraction and management

All articles will be read by 2 reviewers independently, useful data will be extracted from the articles and recorded by Microsoft Excel 2013. Disagreement between the two reviewers will be resolved by discussion or consultation with a third reviewer. For each study, the following data will be extracted: the first author, year of publication, country, number of participants, mean age and gender of participants, interventions details in treatment and control groups, duration, main outcomes, additional outcomes and adverse events. The corresponding authors of the included articles would be contacted if the requisite data were ambiguous or insufficient.

#### 2.4.3 Risk of bias assessment

The risk of bias of all included RCTs will be assessed by two reviewers via the Cochrane Handbook for Systematic Reviews of Interventions tool, which contains the following 7 items: random sequence generation (selection bias), allocation concealment (selection bias), blinding of participants and personnel (performance bias), blinding of outcome assessment (detection bias), incomplete outcome data (attrition bias), selective reporting (reporting bias) and others bias. Each item is classified as “Low risk”, “High risk,” or “Unclear risk”.^[19]^ Any disagreement will be resolved through discussion between the two reviewers. When consultation fails to reach an agreement, arbitration will be resolved by discussion or consultation with a third reviewer.

#### 2.4.4 Treatment effect measurement

For dichotomous variables, rate ratio (RR) will be presented. For continuous variables, mean difference (MD) or standardized mean difference (SMD) will be presents. The confidence intervals (CIs) for both dichotomous and continues variables will be set to 95%.

#### 2.4.5 Dealing with missing data

The corresponding authors will be contact through e-mail if the required data exists any insufficient or missing or unclear. If the author cannot be contacted of the missing data cannot be provided, we will analyze the current available data and discuss it as a limitation.

#### 2.4.6 Assessment of heterogeneity

Statistical heterogeneity across the included studies will be tested using *χ*^*2*^ test and quantified by *I*^*2*^ values. *P* value more than .1 and *I*^*2*^ less than 50% will be defined as no significant heterogeneity, fixed-effect model will be adopted for meta-analysis. *P* value less than .1 and *I*^*2*^ more than 50% will be defined as significant heterogeneity, random-effect model will be adopted for meta-analysis.^[20]^ The possible reasons of heterogeneity will be analyzed by sensitivity analysis or subgroup analysis.

#### 2.4.7 Data synthesis and analysis

Data analysis will be conducted by Review Manager 5.3 software from the Cochrane collaboration. We will select a random-effect model or fixed-effect model to pool the data according to the results of heterogeneity test. if *I*^*2*^ < 50% then the fixed-effect model will be applied for data synthesis. Otherwise, the random-effect model will be conducted if the heterogeneity is significant (*I*^*2*^ ≥ 50%). For the dichotomous data, a Mantel-Haenszel (M-H) method will be used to calculate RRs with 95% CI. For continuous data, inverse variance (IV) method will be used to calculate their mean difference (MD) with 95% CI.

#### 2.4.8 Subgroup analysis

If there is significant heterogeneity and the necessary data are available, subgroup analyses will be carried out according to the duration of BSHX treatment, duration or severity of glaucoma and type of concomitant medication.

#### 2.4.9 Sensitivity analysis

We will use sensitivity analyses to investigate the robustness of main decisions made during the review process in order to evaluate the stability of our results. The main decision includes sample size, quality of studies, methodological and missing data.

#### 2.4.10 Publication bias assessment

If there are more than 10 study included, a funnel plot analysis will be drawn to assess the publication deviation.^[21]^ If asymmetry shows in visual examination, Egger test will be conducted for statistical investigation.^[22]^

#### 2.4.11 Trial sequential analysis

Trial sequential analysis (TSA) will be conducted by TSA 0.9.5 software to assess random errors and imprecision. If the cumulative Z-curve crosses the trial sequential monitoring boundary, the level of evidence is sufficient and no further tests are required. If the Z-curve does not cross the boundary and does not reach the required size of information, the evidence for the result is not sufficient.^[23]^ We will conduct TSA to investigate the reliability of the primary outcome.^[24]^ The sample size needs to be included in further trial will be calculated.

#### 2.4.12 Evidence quality evaluation

Grading of Recommendations Assessment, Development and Evaluate system (GRADE) will be used to evaluating quality of evidence.^[25]^ The quality of evidence will be defined as “high”, “moderate”, “low,” and “very low”.

#### 2.4.13 Patient and public involvement

Patient and public were not involved in this meta-analysis.

#### 2.4.14 Ethics and dissemination

Ethical approval is not required for this meta-analysis as we did not use data related to individual patient. The final report of this systematic review will be published in a peer-reviewed scientific journal or at conferences to provide evidence-based medical support on BSHX method treating glaucoma for clinical workers, and dataset will be made freely available.

## 3 Discussion

Glaucoma is one of the most common eye diseases in clinic, which brings great inconvenience and immeasurable pain to the daily life of patients. The onset is insidious, optic nerve injury and visual impairment have appeared when clear diagnosis has been made. More importantly, glaucoma blindness is irreversible, early detection to control intraocular pressure and prevent optic nerve damage is very important.^[26]^ At present, we do not have much choices for glaucoma neuroprotection and the efficacy is limit.^[27]^ TCM may have an advantage in controlling the progression of optic nerve injury, but there are few systematic reviews of TCM prescriptions for treating glaucoma at present.

TCM focuses on the treatment of personalized pattern differentiation, patients with the same disease may be summarized as different TCM patterns accordingly.^[28]^ Kidney deficiency and blood stasis is one of the common patterns of eye disease in TCM which can be treated by BSHX method base on QiJuDiHuang-Wan, a famous formula applied to the treatment eye disease for more than 200 years.^[14–16]^ Nowadays, more and more glaucoma patients are seeking for alternative therapies. However, in the published studies with small sample size of BSHX method, the effectiveness is uncertain and the quality varies. The efficacy and safety of BSHX method in the treatment of glaucoma are still unclear, and there is no relevant meta-analysis, which limits the application of BSHX method. The purpose of this study was to systematically review the existing literature on the treatment of glaucoma utilizing BSHX method. Finally, the reliability and quality of the main results of this meta-analysis will be evaluated through the TSA and GRADE. This systematic review will provide a comprehensive review of the efficacy and safety BSHX method for glaucoma. The evidence from this review may benefit patients with glaucoma and clinicians to apply BSHX method, as well as to the development of relevant clinical guidelines in the treatment of glaucoma.

## Author contributions

**Conceptualization**: Liyuan Wang, Tianyang Yu, He Sun.

**Data curation**: Ruoxi Liu, Ye Liu.

**Formal analysis**: Liyuan Wang, Tianyang Yu.

**Funding acquisition**: Liyuan Wang, He Sun.

**Investigation**: Ruoxi Liu, Ye Liu.

**Methodology**: Liyuan Wang, Tianyang Yu, He Sun.

**Project administration**: He Sun.

**Resources**: Liyuan Wang, He Sun.

**Software**: Liyuan Wang, Tianyang Yu.

**Supervision**: He Sun.

**Validation**: He Sun.

**Writing – original draft**: Liyuan Wang, Tianyang Yu.

**Writing – review & editing**: He Sun.
